# NiMn_2_O_4_ Ceramic with Bi_2_O_3_ as Ablating Aid with Laser Melting Deposition

**DOI:** 10.3390/ma18112571

**Published:** 2025-05-30

**Authors:** Wei Ren, Xianhai Liu, Shujian Ding, Xiang Weng, Guanghui Liu, Weili Wang, Yanhan Yang

**Affiliations:** 1 School of Physical Science and Technology, Northwestern Polytechnical University, Xi’an 710072, China; renwei@xupt.edu.cn (W.R.); dingshujian@mail.nwpu.edu.cn (S.D.); wengxiang@mail.nwpu.edu.cn (X.W.); lghui96@mail.nwpu.edu.cn (G.L.); 2 School of Science, Xi’an University of Posts & Telecommunications, Xi’an 710121, China; liuxianh_link@163.com (X.L.); yangyanhanxupt@sina.com (Y.Y.)

**Keywords:** NiMn_2_O_4_, laser melting deposition, aging, cationic distribution

## Abstract

NiMn_2_O_4_ thermosensitive ceramics using Bi_2_O_3_ as a low-temperature ablating aid were prepared by laser melting deposition. Analyzing the structural, morphological, and electrical properties of the ceramics revealed important roles of Bi_2_O_3_. The room-temperature resistance decreased gradually with the increasing of the Bi_2_O_3_ content, the thermal constant of the ceramics varied from 2870.1 to 3853.2 K, and the activation energy varied from 0.2473 to 0.3320 eV. Furthermore, the alleviation of the aging issue was attributed to the grain growth and the densification of the ceramics due to the addition of Bi_2_O_3_ and the corresponding cationic redistribution. As a result, an optimized resistance drifting (∆R/R = 5.72%) of the ceramic was obtained with the addition of Bi_2_O_3_.

## 1. Introduction

Negative temperature coefficient thermosensitive ceramics typically have their resistance values exponentially decreasing with increasing temperatures. They have been widely applied in industry for applications such as temperature compensation, temperature monitoring, or inrush current suppressing. Among these ceramics, manganese (Mn)-based materials have attracted much attention due to their high sensitivity and fast response to temperature variations [1,2,3,4]. The prototype of Mn-based thermosensitive materials is Mn_3_O_4_, whose conductive properties are modulated by doping metallic cations. Gradually, Mn-based thermosensitive material evolves into a series of multiple metallic cationic oxide systems, like binary (Ni-Mn-O) [1], ternary (Mn-Co-Ni-O) [2], quaternary (Mn-Co-Ni-Al-O) [3], and quinary (Mn-Zn-Ni-Mg-Al-O) [4,5] systems. One goal of designing such complicated material systems is to meet the riotous needs in domestic, military, and extreme environmental scenarios, such as moon exploring, deep-sea mining, and so on. Another goal is to alleviate the aging issue of the Mn-based thermosensitive material. It is well known that when these materials work in air, their resistances usually vary with time, leading to their conductive behavior degrading. Therefore, it is necessary for these materials to design the compositions and carry out aging tests to determine their time-dependent electrical stability.

As one of the emerging three-dimensional printing additive manufacturing technologies, laser melting deposition (LMD) technology is suitable for fabricating various metallic or ceramic workpieces with high flexibility [6,7,8]. For example, compared to traditional methods, printing a ceramic workpiece with a complicated geometric shape by LMD usually requires a shorter preparation cycle with less or even no sintering/annealing treatments [9,10,11]. In our previous work, LMD was firstly used to prepare NiMn_2_O_4_ thermosensitive ceramics [1]. However, the resistance drift ratios of the ceramics with time were more than 10.38% [1], and many large holes were observed inside the laser-ablated NiMn_2_O_4_ ceramics. These large holes formed when powder particulars were spattered out of the NiMn_2_O_4_ powder bed during the laser ablation procedure, and ambient gas immediately filled the vacancies which were left by spattered particulars (i.e., during laser fused deposition, the laser quickly melts/vaporizes the substrate surface spot, causing a small amount of powder to fly off the substrate surface). These holes will seriously affect the conductive behaviors as well as the aging issue of the ceramics since they are very difficult to spill out after the solidification of the ceramics.

In order to alleviate the aging issue of the laser-ablated ceramic and further densify the ceramic by filling the holes, adding an ablating aid (the small amount of materials that do not react with the target material but are capable of lowering the ablating temperature and help to improve the target material’s quality during the laser ablating process) or glass phase to the ceramic is a viable option [12]. Ablating aids usually have a relatively low melting point temperature and do not chemically react with the ceramics. In 2019, Wang et al. prepared Mn_1.1_Co_1.5_Fe_0.4_O_4_ thermosensitive ceramics by the solid-phase reaction method using Bi_2_O_3_ as a sintering aid, reducing the sintering temperature, and improving the microstructure with an optimal aging coefficient of 0.1% [13]. In 2022, Huo et al. improved the properties of Co_2.77_Mn_1.71_Fe_1.10_Zn_0.42_O_8_ by adding Bi_2_O_3_-B_2_O_3_-SiO_2_-ZnO glass solvents, which affected the grain size and the properties of the material [14]. In 2023, Xie et al. found that the band gap, resistivity, and *B*-value of the xBi(Zn_0.5_Ti_0.5_)O_3_-(1-x)(Ba_0.5_Sr_0.5_)TiO_3_ (0.05 ≤ x ≤ 0.20) ceramics increased with the increasing of the Bi(Zn_0.5_Ti_0.5_)O_3_ content when preparing these ceramics by the solid-state method [15]. Based on above reports, the questions are: can ablating aids be used in LMD-prepared Mn-based thermosensitive ceramics and what will be the effects of the ablating aid on the properties of the ceramics? 

Bi_2_O_3_ is one of the frequently used sintering aids. It has two crystalline structures: monoclinic and triclinic phases [16]. Its melting point temperature is above ~810 °C [17], which is much lower than the traditional sintering temperature of NiMn_2_O_4_ ceramics (at or above 1200 °C [13]). In this work, NiMn_2_O_4_ ceramics modified with Bi_2_O_3_ were prepared by the LMD technique. The effects of the Bi_2_O_3_ addition on the structural, morphological, and electrical properties of the ceramics will be investigated and the possible mechanism will be discussed.

## 2. Experimental Methods

### 2.1. Sample Preparation

NiO and MnO_2_ powders (7 g in total, Zhongnuo New Material Technology Co., Ltd., Beijing, China, 99.9% purity) with a molar ratio of 1:2 were weighed using an electronic balance and placed into a beaker. Then, 5 mL of pure alcohol was poured into the beaker to wet the powders. After ultrasonic cleaning the beaker for 30 min, we stirred the alcohol-wetted powders continuously with a glass rod and slowly added 80 mL of saturated oxalic acid solution to allow the oxalic acid to fully react with the powder until a mixed slurry formed. The beaker was then placed in a water bath pot at 80 °C while the slurry in the beaker was continuously stirred until a viscous paste formed. Then, the beaker was taken out of the water bath pot and put into an oven to dry the paste at 80 °C for 12 h. Subsequently, the dried paste was annealed in a muffle furnace at 800 °C for 2 h. The paste-drying process was necessary since it could reduce the humidity of the slurry and consequently prevent excessive water vapor shortening the service life of the furnace. After annealing processing, spherical-like NiMn_2_O_4_ powder was obtained. Then, the ablating aid, Bi_2_O_3_ powder, with a 99.9% purity purchased from Zhongnuo New Material Technology Co., Ltd., and NiMn_2_O_4_ powder (Bi_2_O_3_ at nominal contents of 0, 0.5, 1.0, and 1.5 wt%, respectively [13]) were uniformly mixed by a planetary ball mill for 5 h to obtain four Bi_2_O_3_-NiMn_2_O_4_ hybrid powders (corresponding to different contents of Bi_2_O_3_).

To perform LMD ablation, a stainless steel plate (5 × 10 cm^2^) was chosen as the substrate with the Bi_2_O_3_-NiMn_2_O_4_ hybrid powder covering the substrate surface into a 2 mm thick layer, i.e., powder bed. An LMD apparatus (model EB-DDLF-4000A, Suzhou Changguang Huaxin Optoelectronics Technology Co., Suzhou, China) was used to ablate the hybrid powder bed and allow the melted powder to naturally cool down to solidify into the ceramic. The ablation was executed by a laser beam track scanning towards one direction at a time. The laser power was set to 1500 W, the scanning speed was 9.72 mm/s, and the laser spot diameter was 2 mm. The distance between every two laser tracks was kept at 7~10 mm in order to prevent the overlapping between laser tracks. The NiMn_2_O_4_ ceramics with different contents of Bi_2_O_3_ (corresponding to the nominal contents of 0, 0.5, 1.0, and 1.5 wt%) were labeled as H1, H2, H3, and H4.

### 2.2. Characterization Methods

The phase composition of the ceramics was identified using a Cu *Kα* X-ray diffractometer (XRD, Bruker D8 Discover, Bruker, Berlin, Germany). Raman spectra were measured by a Raman spectrometer (Finder Vista, Zolix, Beijing, China) in backscattering configuration (with an output power of 20 mW and an incident light wavelength of 532 nm). Scanning electron microscope (SEM, JSM-IT700HR, JEOL Ltd., Tokyo, Japan) was used to examine the surface morphology of the ceramics. The chemical valence states of the surface elements of the ceramics were analyzed using X-ray photoelectron spectroscopy (XPS, Axis UltraDLD, SHIMADZU Co., Kyoto, Japan). Annealing was carried out in air using an annealing furnace (KF1100, Kogent Materials Technology Co., Ltd., Beijing, China). The resistance of the ceramics was measured using a digital multimeter (VICTOR VC97, VICTOR, Shenzhen, China) in an aging test.

## 3. Results and Discussion

**Figure 1:**
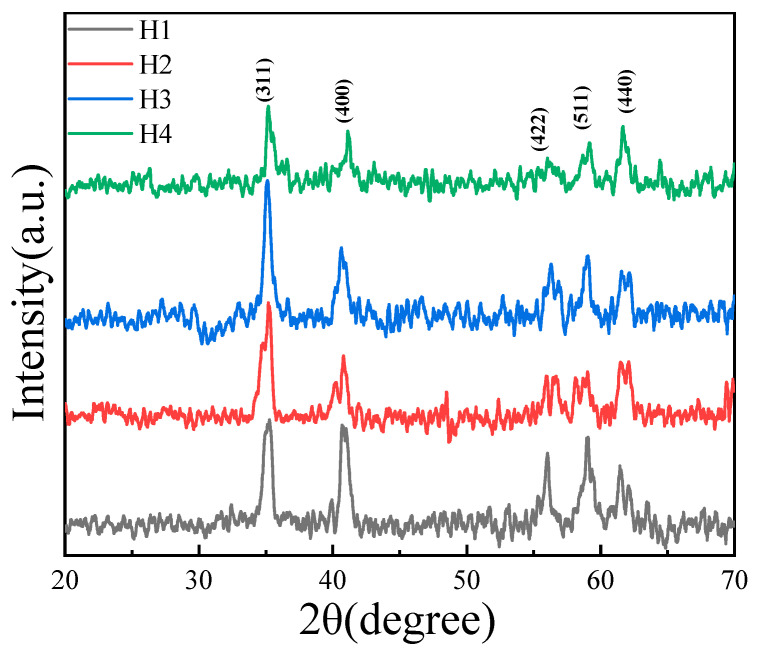
XRD patterns of NiMn_2_O_4_ ceramics with different Bi_2_O_3_ contents. H1, H2, H3, and H4 refer to the samples with the Bi_2_O_3_ contents of 0, 0.5, 1.0, and 1.5 wt%, respectively.

Figure 1 shows the XRD spectra of NiMn_2_O_4_ ceramics with different Bi_2_O_3_ contents. All five diffraction peaks, (311), (400), (422), (511), and (440), are typical spinel peaks, indicating that the ceramics are spinel structured. The ablating aid phase does not affect the phase composition of the ceramics [12]. According to reference [17], the trivalent Bi cation does not tend to enter the spinel lattice. Instead, Bi_2_O_3_ tends to melt and form into a liquid phase during the laser ablation procedure. The existence of liquid-phase Bi_2_O_3_ at the NiMn_2_O_4_ grain boundary regions creates a tight bonding among NiMn_2_O_4_ grains, which facilitates grain growth as well as atomic/cationic mobility [17]. Since the amount of Bi_2_O_3_ is very small, the XRD technology cannot identify the Bi_2_O_3_ phase.

Based on the strongest (311) peaks, the averaged grain sizes of four ceramics can be calculated by following Scherrer’s formula [1]:

(1) $$D=\frac{K\lambda }{\beta cos\theta }$$

where *D* is the grain size, *K* is the Scherrer constant, *λ* is the diffraction wavelength of the X-rays, *β* is the full width at half maximum values of the (311) diffraction peaks, and *θ* is the diffraction angle. The average grain size of the four ceramics can be calculated as 13.44 nm, 13.65 nm, 14.22 nm, and 18.47 nm, respectively. This phenomenon can be explained as following: since the enhanced kinetic energy of grain boundary atoms/cations are caused by the liquid-phase Bi_2_O_3_, small grains tend to disappear or merge into large grains [17]. That is why the addition of Bi_2_O_3_ into NiMn_2_O_4_ ceramics can facilitate the grain growth of the ceramics.

**Figure 2:**
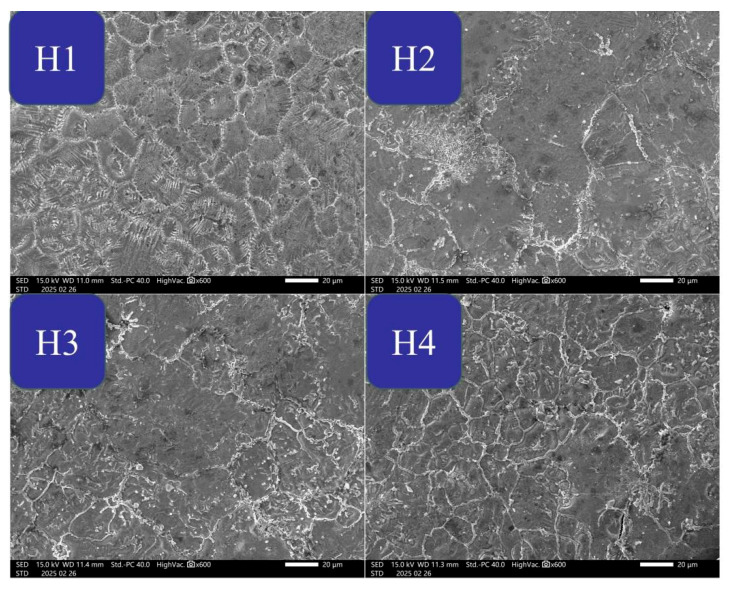
SEM images of NiMn_2_O_4_ ceramics with different Bi_2_O_3_ contents. H1, H2, H3, and H4 refer to the samples with the Bi_2_O_3_ contents of 0, 0.5, 1.0, and 1.5 wt%, respectively.

Figure 2 shows the SEM images of NiMn_2_O_4_ ceramics with different Bi_2_O_3_ contents. The surfaces of the ceramics are very flat and dense for H1~H3, except for the H4 surface where small cracks and holes are observed. Furthermore, without the addition of Bi_2_O_3_ (H1), the surface contains plenty of fine and parallel wrinkles distributed along the particle boundaries; with the addition of Bi_2_O_3_ (H2 and H3), the surface contains several super-large particles and many small particles. This phenomenon is another bit of evidence that the addition of Bi_2_O_3_ facilitates the grain growth of the ceramics. However, the H4 surface is similar to the H1 surface and probably indicates that the excessive addition of Bi_2_O_3_ may not help the NiMn_2_O_4_ grain growth, which is due to the fact that the excessive ablation aid forms a thin liquid layer which separates the particles and prevents the particles merging into a larger one [17].

**Figure 3:**
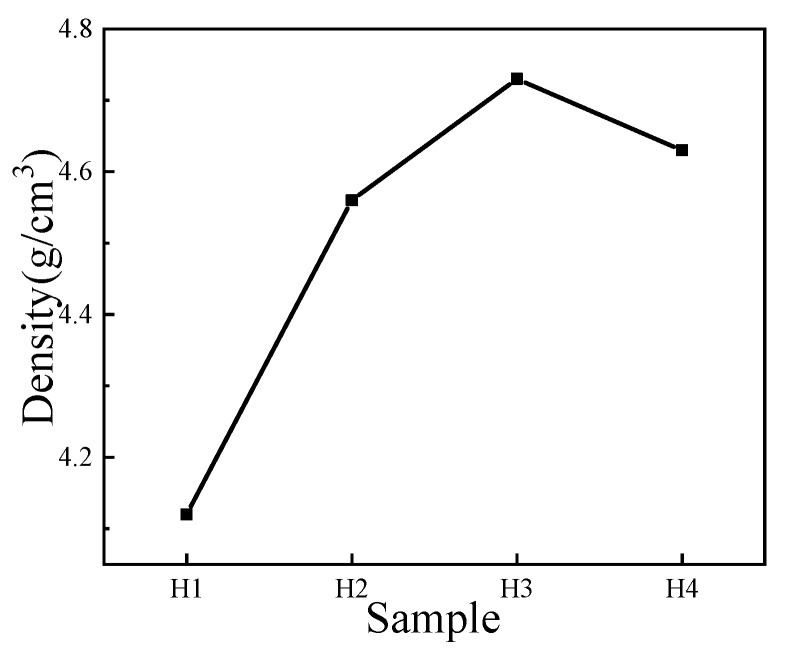
The density (black square) variation in NiMn_2_O_4_ ceramics with different amounts of Bi_2_O_3_. H1, H2, H3, and H4 refer to the samples with the Bi_2_O_3_ contents of 0, 0.5, 1.0, and 1.5 wt%, respectively.

The density of NiMn_2_O_4_ ceramics is mainly affected by laser-ablation-induced holes [1]. The variation due to the density difference between NiMn_2_O_4_ (5.48 g/cm^3^) and Bi_2_O_3_ (8.55~8.9 g/cm^3^) can be simply ignored since the amount of Bi_2_O_3_ is minute. Then, two batches of samples with different Bi_2_O_3_ contents were prepared, and the density of each sample was tested three times. The results from different batches and measurements were averaged and are shown in Figure 3 for the density variation in the NiMn_2_O_4_ ceramics with different amounts of Bi_2_O_3_. When small amounts of Bi_2_O_3_ (H2) and, later, an optimized amount of Bi_2_O_3_ (H3) are added, the density of NiMn_2_O_4_ ceramics increases from 4.12 to 4.74 g/cm^3^, indicating the laser-ablation-induced holes were reduced greatly. The reason is that during laser ablating processing, Bi_2_O_3_ turns into a liquid phase firstly due to its relatively lower melting point temperature (~810 °C) and facilitates the grain growth of NiMn_2_O_4_ ceramics [13]. In addition, with the aid of the liquid Bi_2_O_3_, the NiMn_2_O_4_ grains (from H1 to H2) easily undergo rearrangement [13], which reduces the number of small holes or shrinks the volume of large holes. Correspondingly, the density of the ceramic is improved. With more Bi_2_O_3_ added (from H2 to H3) into the ceramic, the grain growth mechanism and grain rearrangement mechanism are balanced, which further reduces the number of small holes or shrinks the volume of large holes, and the density of the ceramics rises to the maximum value. However, if adding excessive Bi_2_O_3_ (H4), the liquid phase increase further raises the rate of grain growth exacerbating the size difference of the grains. This is primarily because when the temperature increases to 1050 ℃ some closed pores appear; then the higher sintering temperature makes grain boundaries’ motion velocity faster than pores. Therefore, the grain boundary is separated from the pores, and some pores trapped in the grains with the grain grow up further. That is to say, the grain coarsening causing more cracks or holes can not be fully compensated by grain rearrangement. Therefore, the density of the H4 ceramic decreases.

**Figure 4:**
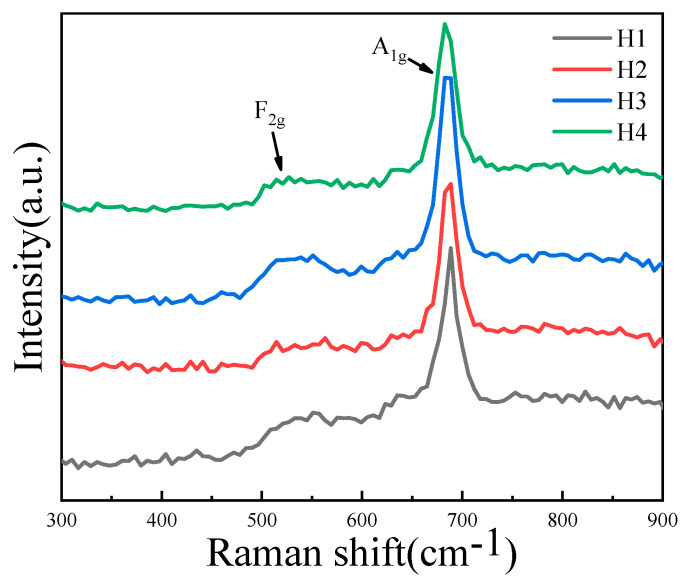
Raman spectra of NiMn_2_O_4_ ceramics with different Bi_2_O_3_ contents. H1, H2, H3, and H4 refer to the samples with the Bi_2_O_3_ contents of 0, 0.5, 1.0, and 1.5 wt%, respectively.

Figure 4 shows the Raman spectra of NiMn_2_O_4_ ceramics with different Bi_2_O_3_ contents. Two absorption peaks are observed at 530 and 683 cm^−1^. For the Mn-based thermosensitive materials, the 530 cm^−1^ Raman peaks are attributed to the symmetric bending vibration of Mn^4+^-O^2−^ (i.e., F_2g_ vibrational modes). The 683 cm^−1^ Raman peaks are due to the symmetric stretching vibration of Mn^3+^-O^2−^ in the octahedral MnO_6_ (i.e., A_1g_ vibrational modes [18]). For H1~H4, the relative intensity ratios of the two peaks do not greatly change, indicating that the ratios of Mn^3+^/Mn^4+^ contents are not affected by the addition of the Bi_2_O_3_ contents. This result is consistent with the above XRD results shown in Figure 1.

**Figure 5:**
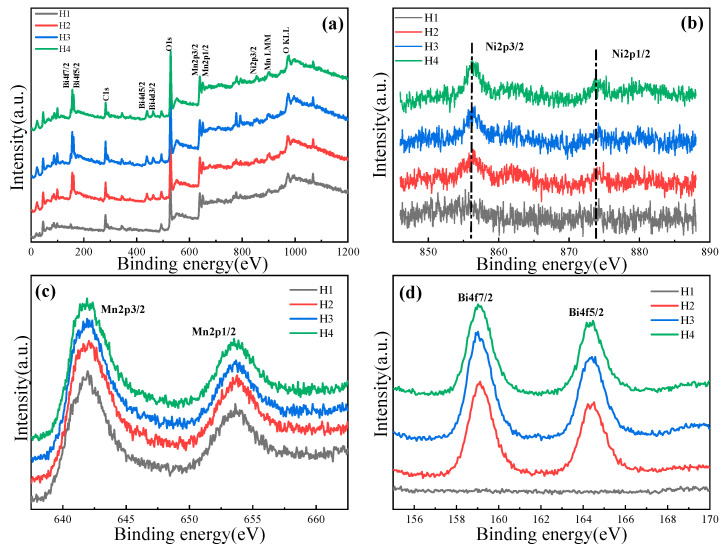
(**a**) Full XPS spectra and high resolution, (**b**) Ni 2p, (**c**) Mn 2p, and (**d**) Bi 4f XPS spectra of NiMn_2_O_4_ ceramics with different Bi_2_O_3_ contents. H1, H2, H3, and H4 refer to the samples with the Bi_2_O_3_ contents of 0, 0.5, 1.0, and 1.5 wt%, respectively.

In order to obtain a detailed cation distribution of the ceramics, XPS spectra were obtained. Figure 5a shows the full XPS spectra of the four ceramics, indicating the existence of the elements Ni, Mn, O, C, and Bi. The fine spectra of Ni, Mn, and Bi were calibrated by a standard C1s peak (248.5 eV) to eliminate the charge effects. Figure 5b shows the Ni 2p energy level spectra where the center positions of the Ni 2p3/2 peak for H1~H4 are located at 856.1 eV, and the center positions of Ni 2p1/2 peak at ~874.4 eV. And the spin–orbit splitting energy between the Ni 2p1/2 peak and the Ni 2p3/2 peak is ~18 eV. These results confirm that Ni cations mainly exist in the valance state of Ni^+2^ [19,20,21]. Figure 5c shows the Mn 2p energy level spectra, where two peaks are observed at ~642.1 and 653.2 eV, corresponding to the Mn 2p3/2 orbitals and Mn 2p1/2 orbitals, respectively, with a spin–orbit splitting energy of about 11 eV. Figure 5d shows the Bi 4f energy level spectra. Among these spectra, the Bi 4f peaks are not detected in the H1 ceramic because Bi_2_O_3_ was not added. The peaks from Bi 4f7/2 and Bi 4f5/2 are identified at the binding energies of 159.1 eV and 164.4 eV, respectively, indicating that a Bi cation exists in the valance state of Bi^+3^ [22,23].

**Figure 6:**
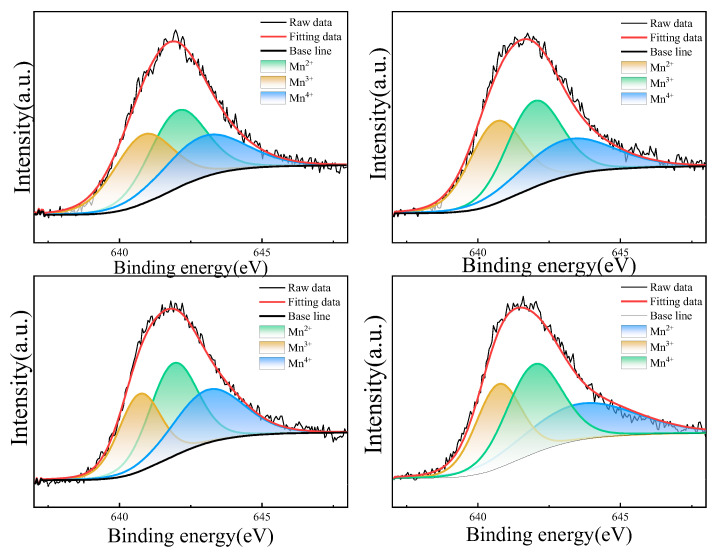
The fitted Mn 2p3/2 XPS peaks of NiMn_2_O_4_ ceramics with different Bi_2_O_3_ contents. H1, H2, H3, and H4 refer to the samples with the Bi_2_O_3_ contents of 0, 0.5, 1.0, and 1.5 wt%, respectively.

Figure 6 shows the fitted curves of the Mn 2p3/2 spectra of the NiMn_2_O_4_ ceramics. The Mn 2p3/2 orbital spectra are chosen for peak fitting because of their good accuracy for the chemical valence state and content distribution of Mn cations [24]. Each fitted spectrum consists of three characteristic sub-peaks, and the centers of the three sub-peaks are located at ~640.7, 641.9, and 643.1 eV, corresponding to Mn^2+^, Mn^3+^, and Mn^4+^, respectively. The Mn cationic contents on the ceramic surface are proportional to the areas of the three sub-peaks. After integrating the areas of the three sub-peaks, the contents of Mn^2+^, Mn^3+^, Mn^4+^, and the Mn^3+^/Mn^4+^ ratios of the H1~H4 ceramics are shown in Table 1.

According to the table, Mn^3+^ contents remain relatively stable at about 38% throughout all the ceramic surfaces. When only a small amount of Bi_2_O_3_ (0.5 wt%) was added, the Mn^2+^ content of the H2 surface increased from 33.79% to 35.02%, while the Mn^4+^ content slightly decreased from 27.67% to 27.09%. This phenomenon indicates that the Mn^2+^ and Mn^4+^ contents are very sensitive to the addition of Bi_2_O_3_. When more Bi_2_O_3_ (1.0 wt%) was added, the Mn^2+^ content of H3 surface sharply decreased from 35.02% to 28.54%, while the Mn^4+^ content greatly increased from 27.09% to 32.44%. This phenomenon indicates that the oxidation state of the H3 ceramic was improved. However, when 1.5 wt% Bi_2_O_3_ was added, the oxidation state of the H4 ceramic was slightly degraded. It is well known that the conductive behavior of the thermosensitive ceramic is determined by the Mn^3+^/Mn^4+^ ratio. In Table 1, the Mn^3+^/Mn^4+^ ratio varies for H1~H4. Particularly, the ratio is 1.20 for the H3 ceramic, which is the lowest.

**Figure 7:**
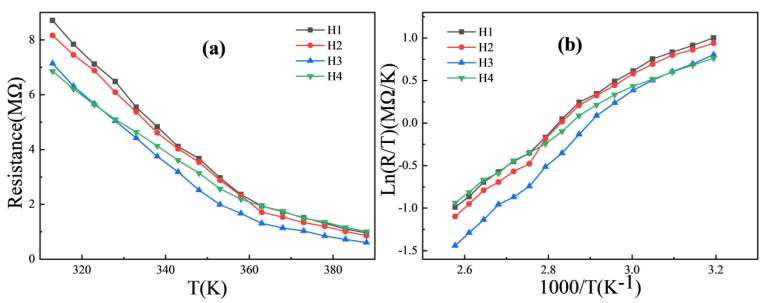
(**a**) *R*-*T*, (**b**) Ln(*R*/*T*)-1000/*T* curves of NiMn_2_O_4_ ceramics with different Bi_2_O_3_ contents. H1, H2, H3, and H4 refer to the samples with the Bi_2_O_3_ contents of 0, 0.5, 1.0, and 1.5 wt%, respectively.

In order to study the variation in the electrical properties of the different ceramics with added Bi_2_O_3_ contents with temperature, the resistance–temperature (*R*-*T* and Ln(*R*/*T*)-1000/*T*) curves of the four ceramics are plotted in Figure 7. In Figure 7a, the resistances of the four ceramics decrease with the increasing temperature, exhibiting a negative temperature coefficient characteristic. The Ln(R/T) vs. 1000/T curves are plotted in Figure 7b. Each curve of H1~H4 roughly conforms to a linear relationship, suggesting that the conductive mechanism of the ceramics is the polaron hopping model [1].

Several key parameters (the resistance *R*, thermal constant *B*, and activation energy *Ea*) are extracted from the curves and shown in Table 2. The *R* values were measured by a digital multimeter, and the *B* value and activation energy *Ea* are calculated by Equations (2) and (3) [1], respectively,

(2) $$B=\frac{Ln(R_{1}/R_{2})}{\left(1/T_{1}\right)-(1/T_{2})}$$

(3) $$E_{a}=B*k$$

where *T*_1_ (313 K) and *T*_2_ (363 K) are two temperature values, *R*_1_ and *R*_2_ are the resistance values corresponding to the two temperature values, and *k* is the Boltzmann constant.

From the table, the room-temperature resistance (*R*_313_) values of the ceramics decrease from 8.71 MΩ (H1) to 6.86 MΩ (H4). This phenomenon can be attributed to the grain growth of the ceramics with the addition of Bi_2_O_3_: the average grain size of H1 is the smallest, and, therefore, the grain boundary region of H1 is the largest. When carriers transport from one spot to another spot in H1, the grain boundary regions scatter the carriers’ directions most frequently, thus leading to the largest ceramic resistance among the four ceramic samples. As for the H2~H4 ceramics, their average grain sizes keep increasing, which further reduces the scattering of the carriers from the grain boundary regions, leading to their room-temperature resistance decreasing.

From the table, the *B* values of the four ceramics increase from 3412.6 to 3853.2 K for H1~H3 and quickly decrease from 3853.2 K (H3) to 2870.1 K (H4), and the *Ea* values increase from 0.2941 to 0.3320 eV for H1~H3 and quickly decrease from 0.3320 eV (H3) to 0.2473 eV (H4). That is, the sensitivity of H3 to the temperature variation is the best. This phenomenon cannot be simply explained by the grain growth. In fact, the appropriate amount of Bi_2_O_3_ can adjust the grain arrangement, resulting in the densification and the improvement of the grain size for the H1~H3 ceramics. The defects (voids or holes inside the ceramic) and the grain boundaries are correspondingly reduced. Therefore, the H3 ceramic bears the maximum *B* value. Although the excessive addition of Bi_2_O_3_ (H4) facilitates the NiMn_2_O_4_ grain growing to the maximum value (18.47 nm), and the grain coarsening induces more defects, which cannot be compensated by the grain arrangement. Therefore, the *B* value of H4 is decreased. In addition, the Mn^3+^/Mn^4+^ ratio of H3 is the closest to one, which is another indication that the electrical properties of the H3 ceramic are better than the other three ceramics.

**Figure 8:**
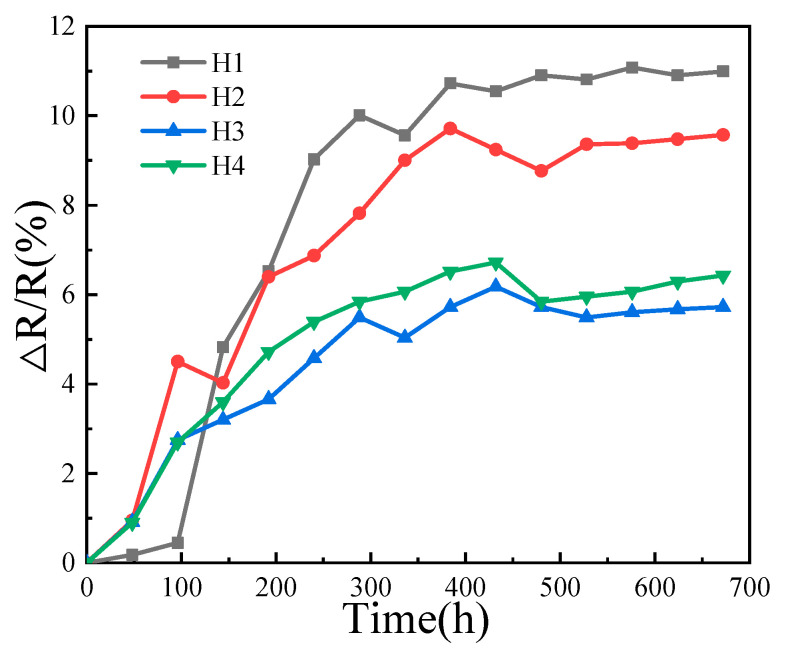
The evolution of the aging rate of NiMn_2_O_4_ ceramics with different Bi_2_O_3_ contents. H1, H2, H3, and H4 refer to the samples with the Bi_2_O_3_ contents of 0, 0.5, 1.0, and 1.5 wt%, respectively.

In the aging test, each of the H1~H4 ceramics was placed in a furnace and annealed in air at 125 °C for about 600 h. The room-temperature resistance was tested every 48 h during annealing, and the resistance drift rate was calculated according to Equation (4) [1].

(4) $$\alpha =∆R/R_{0}=\frac{R-R_{0}}{R_{0}}$$

where *R* is the room-temperature resistance value after the annealing treatment for 600 h; *R_0_* is the initial room-temperature resistance (i.e., before aging test). The aging behavior of the ceramics with different amounts of Bi_2_O_3_ is shown in Figure 8. The aging curves of the four ceramics exhibit point-to-point fluctuations, but the evolution trends can still be obtained. From the figure, the resistance drift rate gradually decreases from H1 (10.99%, which is consistent with our previous results [1]) to H3 (5.72%), which indicates that the electrical stability of the ceramics keeps increasing. However, the resistance drift rate of H4 deteriorates. These phenomena can be explained as follows: for the H1~H3 ceramics, with a greater addition of Bi_2_O_3_, the ceramic densification is gradually improved and the internal defects, such as voids, vacancy, etc., are greatly reduced. As a result, the migration of cationic vacancies during the annealing process becomes more and more difficult and the resistance drift rate of the H3 ceramic decreases to the lowest 5.72% after annealing for 600 h. As for H4, an excessive about of Bi_2_O_3_ was added and facilitates the grain growth of the ceramic. Therefore, a large number of oversized grains form and aggregate (i.e., grain coarsening). Considering that tiny pores may more possibly form among oversized grains, these tiny pores can provide a favorable environment to adsorb residual oxygen (there are plenty of air molecules between the powder particulates before the laser ablation) into the spinel lattice during annealing, and the absorbed residual oxygen reacts with the internal cation, which alters the cationic distribution and thus deteriorates the electrical stability of the ceramics.

**Figure 9:**
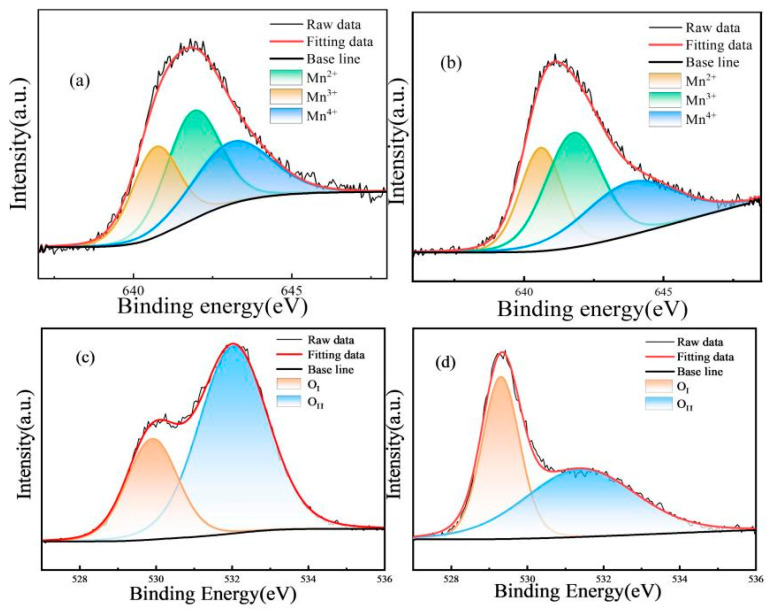
The fitting (**a**) and (**b**) Mn 2p3/2 orbital XPS peaks of H3 before and after aging tests; the fitting (**c**) and (**d**) O 1s orbital XPS peaks of H3 before and after aging tests. H1, H2, H3, and H4 refer to the samples with the Bi_2_O_3_ contents of 0, 0.5, 1.0, and 1.5 wt%, respectively.

In order to further investigate the aging mechanism of the ceramics, the spectra of the Mn2p3/2 orbitals and O 1s orbitals before and after the aging tests of the H3 ceramic were fitted. Figure 9a,b show the fitted sub-peaks of the three valence states of the Mn cations of H3 before and after aging tests. The ratio of Mn^3+^/Mn^4+^ changes from an initial 1.20 to 1.42 after 600 h of the aging test, which indicates a significant change in the Mn cation distribution in the ceramic, particularly coupling with an increase in Mn^3+^. Observing the two sub-peaks of the O 1s orbitals in Figure 9c,d, the peak with the lower binding energy corresponds to the lattice oxygen and the peak with the higher binding energy corresponds to the adsorbed oxygen. After the 600 h aging treatment, the content of the lattice oxygen of the H3 ceramic increases and the content of the adsorbed oxygen decreases. This can still be explained as the adsorption of residual oxygen molecules during the aging test [24]. Correspondingly, the resistance value changes, and the mechanism is described by Equation (5) [24].

(5) $$\frac{1}{2}V_{o}^{..}+\frac{1}{2}O_{2}+{Mn}^{2+}\to {Mn}^{3+}+\frac{1}{2}O^{x}$$

## 4. Conclusions

NiMn_2_O_4_ thermosensitive ceramics with Bi_2_O_3_ as the ablating aid were successfully prepared using the LMD technique. The addition of Bi_2_O_3_ does not affect the spinel phase of NiMn_2_O_4_. Since a suitable amount of Bi_2_O_3_ (i.e., 0.5 and 1.0 wt%) facilitates the grain growth and makes the grains easier to be rearranged, NiMn_2_O_4_ grains gradually grow, the surface morphology becomes flatter, and the NiMn_2_O_4_ ceramics tends to be more dense. However, adding excessive Bi_2_O_3_ (1.5 wt%) would promote the grain growing so fast that some pores reappear, the surface morphology slightly deteriorates, and the density of NiMn_2_O_4_ ceramics is lowered. XPS analyses show that Mn cations (Mn^2+^, Mn^3+^, and Mn^4+^) will vary with Bi_2_O_3_ contents. In particular, the material constant value and resistance drift of the ceramics also vary from 2870.1 to 3853.2 K and from 5.72% to 10.99%, respectively, which were mainly attributed to the adsorption of residual oxygen molecules during the aging test. In summary, Bi_2_O_3_ as a sintering aid during the preparation of NiMn_2_O_4_ ceramics by LMD is a promising method for the optimization of the electrical properties of the thermosensitive ceramics. In the future, other sintering aid materials might be added to further optimize the electrical and ageing properties of the LMD-synthesized thermosensitive ceramics.

## Figures and Tables

**Table 1:** The contents of Mn^2+^, Mn^3+^, Mn^4+^, and the Mn^3+^/Mn^4+^ ratios of the H1~H4 ceramics.

No	Mn^2+^	Mn^3+^	Mn^4+^	Mn^3+^/Mn^4+^
H1	33.79 ± 0.03%	38.54 ± 0.02%	27.67 ± 0.03%	1.39
H2	35.02 ± 0.02%	37.89 ± 0.04%	27.09 ± 0.04%	1.40
H3	28.54 ± 0.04%	39.02 ± 0.01%	32.44 ± 0.04%	1.20
H4	29.77 ± 0.02%	38.80 ± 0.03%	31.43 ± 0.02%	1.23

**Table 2:** *R*, *B*, and *E*a values of NiMn_2_O_4_ ceramics with different Bi_2_O_3_ contents.

No	*R*_313_(MΩ)	*R*_363_(MΩ)	*B*_313/363_(K)	*Ea*(eV)
H1	8.71 ± 0.05	1.94 ± 0.04	3412.6 ± 0.2	0.2941 ± 0.0005
H2	8.16 ± 0.06	1.71 ± 0.05	3551.2 ± 0.3	0.3060 ± 0.0003
H3	7.14 ± 0.04	1.31 ± 0.05	3853.2 ± 0.2	0.3320 ± 0.0005
H4	6.86 ± 0.05	1.94 ± 0.04	2870.1 ± 0.2	0.2473 ± 0.0005

## Data Availability

The original contributions presented in this study are included in the article. Further inquiries can be directed to the corresponding author.
